# Visual attention in mixed-gender groups

**DOI:** 10.3389/fpsyg.2014.01569

**Published:** 2015-01-12

**Authors:** Mary Jean Amon

**Affiliations:** Department of Psychology, Center for Cognition, Action, and Perception, University of CincinnatiCincinnati, OH, USA

**Keywords:** eye tracking, gaze, gender, group dynamics, objectification, social cognition

## Abstract

A basic principle of objectification theory is that a mere glance from a stranger represents the potential to be sexualized, triggering women to take on the perspective of others and become vigilant to their appearance. However, research has yet to document gendered gaze patterns in social groups. The present study examined visual attention in groups of varying gender composition to understand how gender and minority status influence gaze behavior. One hundred undergraduates enrolled in psychology courses were photographed, and an additional 76 participants viewed groupings of these photographs while their point of gaze was recorded using a remote eye-tracking device. Participants were not told that their gaze was being recorded. Women were viewed more frequently and for longer periods of time than men in mixed-gender groups. Women were also more likely to be looked at first and last by observers. Men spent more time attending to pictures of women when fewer women were in the group. The opposite effect was found for pictures of men, such that male pictures were viewed less when fewer pictures of men were in the group. Female observers spent more time looking at men compared to male observers, and male observers spent more time looking at women than female observers, though both female and male observers looked at women more than men overall. Consistent with objectification theory, women's appearance garners more attention and interest in mixed-gender social groups.

## Introduction

The ability to follow another person's point of gaze plays an important role in social cognition. Gaze direction indicates the presence of important objects in the environment. It is intimately tied to the ability for goal-directed action and is thus revealing of one's intentions (see Perrett and Emery, 1994; Baron-Cohen, 1997). The wealth of nonverbal information provided by eye movements, as well as its accessibility, makes eye gaze useful for developing theory of mind, or inferring the mental states of other people. When viewing another person's point of gaze, one can imagine what they are seeing and thinking about (Perrett and Emery, 1994; Baron-Cohen, 1997). Gaze direction may be subtle, but it is a powerful cue that reflexively shifts attention. Even when it is irrelevant to one's task, gaze detection occurs automatically (Langton et al., 2000). An indirect stare orients people to the direction of the averted gaze, while a direct stare captures the attention of the target (Yokoyama et al., 2013). Direct gaze is preferentially processed (Yokoyama et al., 2013), perhaps because it has immediate personal relevance. Being the subject of another's stare may signal interest, friendliness, attraction, dominance, or aggression (Argyle and Cook, 1976; Kleinke, 1986; Shimojo et al., 2003). Regardless of the underlying intention, interpersonal gaze is the earliest signal of potential social interaction (von Grünau and Anston, 1995).

Given the relationship between gaze and social cognition (Macrae et al., 2002), it follows that micro-level cues such as gaze have the potential to influence social outcomes. In particular, objectification theory has outlined the numerous effects of eye gaze on women. Women's objectification is defined by experiences of sexualization, whereby women are regarded as objects or collections of body parts (Fredrickson and Roberts, 1997). A basic principle of objectification theory is that a mere glance from a stranger represents the potential to be sexualized, triggering women to take on the perspective of others and become vigilant to their own appearance. According to this theory, gaze always comes with the potential for sexualization and is among the most ubiquitous form of objectification experienced by women (Fredrickson and Roberts, 1997).

Objectifying gaze, or the “visual inspection of the body by another person,” need not be accompanied by catcalling or leering to negatively affect targets (Gervais et al., 2011). In the absence of explicit sexist remarks, women may still feel “checked out” and evaluated based on their adherence to societal standards of beauty (Fredrickson and Roberts, 1997). “Seeing eye to body” causes women to be perceived as and behave more like objects (Heflick and Goldenberg, 2014). Women talk less, become more passive, and perform worse on math tests when their bodies are salient to others (Saguy et al., 2010; Gervais et al., 2011; Calogero, 2013). The anticipation of male gaze can also induce body shame and social physique anxiety (Calogero, 2004). Even imagined objectification from others can negatively impact women's body satisfaction and self-esteem (Tiggemann, 2001). Without information to the contrary, male gaze demonstrates visual inspection and the potential for objectification (Kaschak, 1992). Within this framework, gaze frequency and duration in social settings may significantly influence women's physical, psychological, and social outcomes (see Calogero et al., 2011, for review).

Preliminary evidence suggests that visual attention is guided in part by gender bias. Early observational research carried out in social dyads indicated that women were looked at more than men, especially by other women (Hall, 1984). Hall's general conclusion from reviewing eye gaze literature was that higher levels of gaze are found in same-sex interactions. However, Hall's review of interpersonal gaze is limited in that it focuses on observational research in social dyads. It also does not outline the contexts in which each of these interactions took place or cite individual studies discussed throughout the book. More recently, research demonstrated that visual attention toward women often entails objectifying gaze. Gervais et al. (2013) were among the first to investigate the nature of objectifying gaze, demonstrating that participants focused more on women's bodies when targets fit cultural beauty ideals and when they were instructed to focus on target appearance, with men exhibiting more objectifying gaze than women observers. Research on perception of women's bodies reflects a shift in the objectification literature, from indirectly investigating women's self-objectification to directly examining the “literal objectification” of women, or the focus on women's physical appearance (Heflick and Goldenberg, 2014).

Despite this shift, most research on interpersonal gaze examines visual attention toward a single target, and the hypothesis that women are looked at more than men has not been examined in social groups. While visual attention in dyads is useful in understanding certain types of social dynamics, it does not describe how gaze unfolds in groups where women may often be “looked at” or “checked out” in passing. Complex environments, such as group settings, may be more likely to elicit a variety of attentional goals, which leads to the greater likelihood for gendered gaze behavior (Duncan et al., 2007). Visual attention in group settings can be used to elucidate processes of social cognition, as well as normative cues of intergroup biases. Gaze behavior also has implications for the experience of targets, particularly women and minorities. The present study is the first to examine visual attention in same- and mixed-gender groups to understand how gender and minority status influence gaze behavior. One hundred undergraduates enrolled in psychology courses were photographed. An additional 76 participants viewed groupings of these photographs while their point of gaze was recorded using a remote eye-tracking device. Participants were not told that their point of gaze was being recorded until the experiment ended.

*Hypothesis 1.* In line with research suggesting that both men and women objectify women (e.g., Gervais et al., 2013), it was hypothesized that female and male observers would view pictures of women more frequently and for longer periods of time compared to pictures of men. In line with the assumption that observers would focus more on pictures of women, it was expected that women would be more likely to be looked at first and last across trials. While female observers were expected to look more at pictures of women overall, I further predicted that female observers would look at pictures of men more than male observers would. This prediction follows from sociosexual theories that suggest people scan for potential mates or compatible partners for interaction (e.g., Duncan et al., 2007).
*Hypothesis 2.* Group gender ratio was also expected to influence gaze behavior and reinforce findings that women are particularly attractive targets of gaze. An observer's focus on female pictures was expected to increase as the ratio of women on the screen decreased, such that women would draw more attention as minority group members. In contrast, it was expected that pictures of men would not attract more visual attention when men were the minority.
*Hypothesis 3.* I assumed that male observers would display more gaze aversion, or more time looking away from pictures of men and women, in trials with more male pictures than female pictures. This prediction follows from heterosexual norms of “girl watching” (Quinn, 2002) and objectification theory, which suggest that women are considered more appropriate targets of gaze than men.
*Hypothesis 4.* Lastly, I hypothesized that men would be relatively accurate in predicting their own gendered gazing and would report looking at women more than men in survey measures. Women were predicted to report looking at men more than women, and this was expected to contradict their focus on pictures of women. This follows from the assumption that men and women would report gazing behavior consistent with heterosexual norms.

## Methods

### Participants

Undergraduate students in psychology classes were enrolled into a two-part study via an online recruitment system. Fifty female and 50 male participants completed the first phase of the study (*N* = 100). Participants ranged in age from 18 to 26, with a median age of 19. Eighty percent of participants identified as White, 12% as biracial, 7% as Black, and 1% as Asian. Two women were bisexual and three male participants were homosexual or bisexual. Fifty-five percent of participants reported that they were not in a committed relationship.

An additional 39 women and 37 men participated in the second portion of the experiment (*N* = 76). Participants ranged in age from 18 to 48, with a median age of 19. Eighty-two percent were White, 7% were Black, 5% were Asian, 4% were biracial, and 3% were Hispanic. Participants were predominantly heterosexual, with the exception of three bisexual women and one homosexual man. The majority of participants were single, with 57% reporting that they were not in a committed relationship.

### Procedure

#### Part 1

The first group of participants responded to a study broadly described as examining beliefs about the opposite sex. Upon obtaining informed consent, participants completed a demographic form. Participants were then asked to take off easily removable accessories and their clothing was covered with a cape. They stood in front of a white background and maintained a neutral face while looking into the camera. Photographs were taken from the shoulders-up. Participants were then debriefed and thanked for their time.

#### Part 2

A second group of participants was recruited into a study described as investigating physiological responses to visual stimuli. In order to reduce social desirability response biases and elicit more natural gaze responses from participants, social cognition tasks were initially described to participants as measuring tear production (rather than gaze) and heart rate. Participants were also told that they would be randomly assigned to see pictures of art, landscapes, animals, people, or cartoons. In reality, all participants viewed photographs of Part 1 participants while their point of gaze was recorded.

After obtaining consent, participants were seated in front of a computer monitor, and they were calibrated to a remote eye-tracking device using a nine-point configuration. Point of gaze was measured as participants viewed 75 screens of Part 1 photographs that systematically varied in group size and gender composition. Following the eye tracker portion of the study, participants answered questions about their gaze behavior and completed a demographic form. All research was carried out in accordance with the protocol approved by the University of Cincinnati's Institutional Review Board.

### Materials

#### Equipment and stimulus

Part 1 participant photographs were cropped and adjusted for consistency in size and shading. Pictures were 3.5 × 3.5 inches and presented in color. Part 2 participants viewed these photographs while eye-tracking data was collected via an Applied Science Laboratory D6 remote eye-tracking device, which captured corneal reflections to calculate the real-time point of gaze trajectory. Calibration of the camera was established using a 9-point grid, and point of gaze trajectories were recorded at 60 Hz.

Trials with photos of all women or all men represented baseline conditions to compare how pictures of women and men are viewed in gender-homogenous groups. Mixed-gender trials tested the effect of group size and gender composition on visual attention. Fifteen trial types were displayed, each with a set number of photographs and gender ratio. Each trial type was displayed five times, such that Part 2 participants viewed 75 stimulus display screens in total. While there was a fixed gender ratio within each trial type, female and male pictures were randomly assigned to each trial and screen space. The order of trials was also randomized. Pictures of racial minorities were randomly drawn into trials based on gender. Of the women pictured, three identified as African-American and five were biracial. Of the men who were pictured, four identified as African-American, six were biracial, and one was Asian. Given the small number of Part 1 participants who were racial minorities, race was not included as a primary variable in analyses. Screens had one, two, four, or six pictures. In the one-picture trial, either a woman or man's photo was shown (1:0 ratio). The two-picture trial had two women (2:0), two men (2:0), or one woman and one man (1:1). The four-person group presented all women (4:0), all men (4:0), one woman and three men (1:3), or one man and three women (1:3). The six-picture trial was composed of all women (6:0), all men (6:0), one woman and five men (1:5), one man and five women (1:5), two women and four men (2:4), and two men and four women (2:4).

In one-picture conditions, the photograph was centered on the screen. In two-picture conditions, the photographs were next to each other. Two rows of pictures were presented in the four-picture and six-picture trials, with two and three pictures on each row, respectively. When multiple pictures were presented, they were spaced 1.75 inches apart from one another on all sides and centered as a grouping on the screen. Each trial was presented for 10 s each, with 2 s gaps in between trials.

### Measures

Raw data points were aggregated within each trial based on target of gaze, i.e., female picture, male picture, or background area. Gaze duration, frequency, and sequence of viewing were calculated for each of the 75 trials.

#### Gaze duration

Time during which point-of-gaze was captured within the boundary of a picture region was totaled for each picture viewed during the 75 trials to derive a gaze duration score. These scores were standardized prior to analysis. The average time participants looked at blank screen space during a trial set was subtracted from the 10 s trial time. The average time participants looked at pictures, vs. blank screen space, was divided by the number of pictures in the trial set. The resulting number was a baseline for how long the average picture was looked at during a trial set with one, two, four, or six pictures, accounting for the time each participant attended to blank screen space. Next, this number was subtracted from the time each picture of a woman and man was viewed during a trial. If the number was positive, the picture was gazed at more than average. If negative, the picture was gazed at less than average. Times spent viewing women and men were averaged within each trial type to determine mean time spent gazing at women and men in different group settings. An increase in gaze duration is associated with targets that are deemed particularly interesting in the environment, both in terms of importance and visual attractiveness (Findlay et al., 1995).

#### Gaze frequency

Gaze frequency was defined as the number of times point-of-gaze fell within the boundaries of a picture after having been outside of the picture boundaries during a trial. Both visual searches, when participants briefly scanned across a picture, and fixations were included in gaze frequency count. For example, if a picture was viewed once during the trial and returned to one more time during the same trial, the picture would have a gaze frequency of two regardless of how long the picture was viewed. Gaze frequency was used as an additional measure of attention toward male and female targets. While both gaze duration and frequency are commonly used measures of attention, it has been suggested that gaze frequency is an indicator of attentional engagement and duration an indicator difficulties disengaging (Yiend et al., 2013).

#### Gaze sequence

Sequence data identified the order in which pictures were observed during each trial in order to determine whether men or women were more likely to be looked at first and last. Because of rapid orienting at the beginning of each trial, the number of times female and male pictures were looked at in the first five gaze sequences was totaled. The last five gaze sequences were also totaled to determine the gender of targets viewed last. The first pictures viewed measured initial interest. Last looks were a measure of sustained attention and indicated the pictures that observers settled on toward the end of each trial.

#### Gaze avoidance

Gaze avoidance was calculated for each trial type by averaging time spent looking at blank screen space, outside of the bounds of pictures. Time during which point-of-gaze fell outside of the boundaries of pictures was aggregated for each trial and then averaged across trial type. Previous studies have suggested that personality traits such as inhibition, mental illness, and self-esteem may impact gaze aversion (e.g., Larsen and Shackelford, 1996; Vandromme et al., 2011). In the present study, gaze avoidance was used to examine the lack of interest in male and female targets or, alternatively, discomfort in viewing targets.

#### Beliefs about social gaze

All participants completed a demographic information form. Part 2 participants also answered questions assessing beliefs about their gaze behavior. Six items were rated along a seven-point scale (1 = *strongly disagree*; 7 = *strongly agree*). Participants indicated their agreement with the statements, “I often gaze at women,” “I often gaze at men,” “women often look at me,” “men often look at me,” “I pay attention to attractive and beautiful people,” and “I pay attention to unattractive people.”

## Results

### Gaze duration

A series of 2 (observer gender) × 2 (target gender) mixed-design analyses of variance (ANOVA) were conducted to evaluate the hypotheses that observers would view pictures of women for longer periods of time compared to pictures of men and female observers would view pictures of men more than male observers. Trials with similar gender ratios were compared to determine how female and male targets are looked at under similar conditions. For example, trials with one female and five male pictures were compared to those with one male and five female pictures. Findings revealed that observers did not differ in how long they looked at women and men in baseline trials comparing time spent viewing female or male pictures in gender-homogenous groups. This was true for all main effects and interactions in baseline trials, including those with one, two, four, or six pictures (*p* > 0.05).

**Figure 1:**
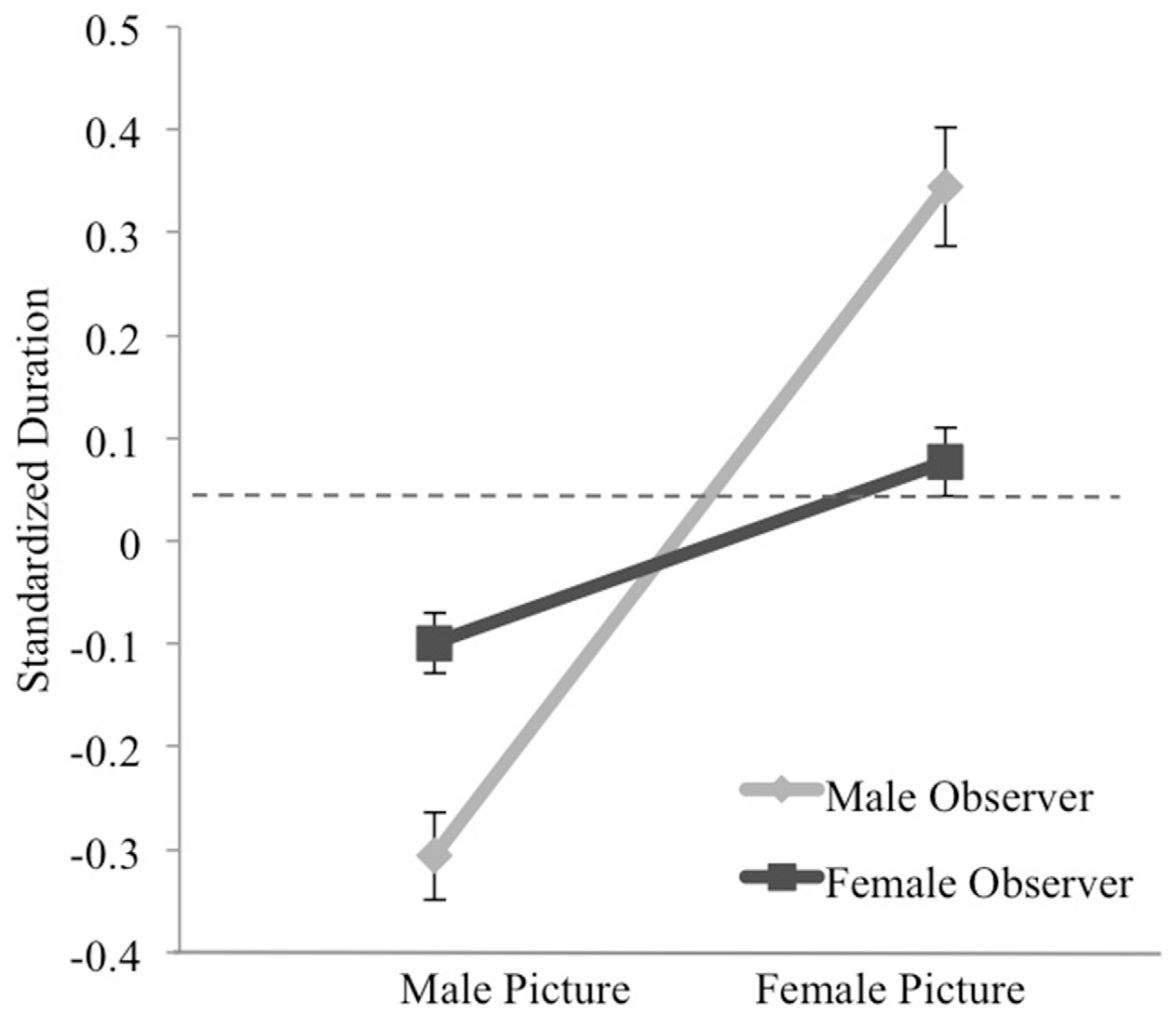
**A significant interaction demonstrated that female and male observers viewed pictures of women for longer periods of time across mixed-gender trials**. In addition, female gazers looked at male pictures more than male gazers, and male gazers looked at female pictures more than female gazers.

Average time female and male participant pictures were viewed during mixed-gender trials was compared. A 2 (observer gender) × 2 (target gender) ANOVA analyzing gaze duration toward women and men across all mixed-gender groupings uncovered a non-significant main effect of observer gender, a significant effect of target gender, and a significant interaction between observer and target gender (see Table 1 for summary of results and Table 2 for means and standard deviations). The interaction demonstrated that female observers spent more time viewing male pictures than male observers while male observers spent more time viewing female pictures than female observers, though female pictures were viewed for longer periods of time by women and men overall (see Figure 1).

**Table 1:** **Summary of results from 2 × 2 mixed-design ANOVAs examining standardized gaze duration scores in mixed-gender trials**.

**Trial type**	***F***	***p***	**η^2^_*p*_**
**ALL MIXED-GENDER TRIALS**			
Observer gender	3.36	0.07	0.04
Gender ratio	52.23^***^	<0.001	0.41
Observer × gender ratio	17.24^***^	<0.001	0.19
**1:1 WOMAN OR MAN**			
Observer gender	1.26	0.27	0.02
Gender ratio	13.15^***^	0.001	0.15
Observer × gender ratio	4.90^*^	0.03	0.06
**3:1 MAJORITY WOMAN OR MAN**			
Observer gender	1.05	0.31	0.01
Gender ratio	41.16^***^	<0.001	0.36
Observer × gender ratio	9.10^**^	0.003	0.11
**1:3 MINORITY WOMEN OR MEN**			
Observer gender	1.49	0.23	0.02
Gender ratio	50.30^***^	<0.001	0.41
Observer × gender ratio	11.22^***^	0.001	0.13
**5:1 MAJORITY WOMEN OR MEN**			
Observer gender	0.03	0.87	<0.001
Gender ratio	15.40^***^	<0.001	0.17
Observer × gender ratio	19.45^***^	<0.001	0.21
**4:2 MAJORITY WOMEN OR MEN**			
Observer gender	0.01	0.92	<0.001
Gender ratio	21.05^***^	<0.001	0.22
Observer × gender ratio	8.47^**^	0.005	0.10
**2:4 MINORITY WOMEN OR MEN**			
Observer gender	3.57	0.06	0.05
Gender ratio	33.28^***^	<0.001	0.31
Observer × gender ratio	5.96^*^	0.02	0.08
**1:5 MINORITY WOMEN OR MEN**			
Observer gender	4.40^*^	0.04	0.06
Gender ratio	24.33^***^	<0.001	0.25
Observer × gender ratio	13.14^***^	0.001	0.15

ANOVAs compared how long men and women were viewed in similar group settings.

Significance:

* 0.05,

** 0.01,

*** 0.001.

**Table 2:** **Means and standard deviations for standardized gaze duration scores between men and women observers toward same- and opposite-sex targets**.

**Picture ratio**	**Observer gender**	**Female picture**		**Male picture**	
		***M***	***SD***	***M***	***SD***
All mixed-gender	Female	0.077	0.21	−0.10	0.18
	Male	0.34	0.35	−0.31	0.26
1:0	Female	0.18	0.73	0.18	0.73
	Male	−0.06	0.91	−0.01	0.96
2:0	Female	0.01	0.42	−0.07	0.34
	Male	−0.04	0.44	−0.07	0.38
1:1	Female	0.10	0.74	−0.15	0.66
	Male	0.40	1.05	−0.64	0.96
4:0	Female	−0.03	0.15	0.03	0.15
	Male	0.001	0.19	−0.01	0.18
3:1	Female	0.09	0.20	−0.06	0.20
	Male	0.18	0.26	−0.23	0.34
1:3	Female	0.18	0.54	−0.24	0.44
	Male	0.65	0.86	−0.51	0.49
6:0	Female	−0.006	0.09	−0.02	0.08
	Male	0.02	0.09	−0.009	0.13
5:1	Female	−0.02	0.14	−0.007	0.12
	Male	0.09	0.15	−0.13	0.19
4:2	Female	0.06	0.17	−0.02	0.21
	Male	0.16	0.21	−0.13	0.23
2:4	Female	0.08	0.37	−0.16	0.21
	Male	0.32	0.44	−0.21	0.33
1:5	Female	0.06	0.56	−0.07	0.47
	Male	0.57	0.66	−0.28	0.43

Planned comparisons examined subsources of variation among different mixed-gender groups. The pattern found across mixed-gender groups was consistent across 2 (observer gender) × 2 (target gender) ANOVAs examining two-picture, four-picture, and six-picture mixed-gender configurations, including those comparing 1:1, 3:1, 1:3, 5:1, 4:2, and 2:4 ratios. An exception to this pattern was seen in a 2 × 2 ANOVA examining trials with either one woman or one man in six-picture trials, where both main effects were significant. However, similar to other mixed-gender trials, the interaction revealed that pictures of women were viewed for longer periods of time than pictures of men, with male observers viewing pictures of women more than female observers and female observers viewing pictures of men more than male observers.

### Gaze duration based on majority and minority status

#### Female gender ratio

Mixed-design ANOVAs tested duration of time female pictures were viewed by female and male observers across trials with the same number of pictures but different gender ratios. It was hypothesized that the fewer the women in the group, the longer the individual women would be viewed. A 2 (observer gender) × 2 (gender ratio) ANOVA comparing gaze duration during two-picture trials with either a 1:1 or 2:0 gender ratio revealed a significant main effect of trial type, *F*_(1, 74)_ = 5.98, *p* = 0.02, η^2^_*p*_ = 0.08. Individual pictures of women were viewed longer when there was one female picture (*M* = 0.24, *SD* = 0.91) compared to two pictures (*M* = −0.01, *SD* = 0.43). Female and male observers were not significantly different in time spent looking at female pictures, nor was the interaction between observer gender and gender ratio significant, *p* > 0.05.

A 2 (observer gender) × 3 (gender ratio) ANOVA testing gaze duration toward female pictures in four-picture trials revealed significant main effects of observer gender, *F*_(1, 74)_ = 9.07, *p* = 0.004, η^2^_*p*_ = 0.11, and gender ratio, *F*_(2, 148)_ = 19.89, *p* < 0.001, η^2^_*p*_ = 0.21, and an interaction of gender and ratio, *F*_(2, 148)_ = 6.21, *p* = 0.003, η^2^_*p*_ = 0.08. Men spent significantly more time viewing female pictures than women did (*M* = 0.07, *SD* = 0.09; *M* = 0.02, *SD* = 0.05), though female and male gazers viewed pictures of women longer than pictures of men. The simple effect of gender ratio for male observers violated the sphericity assumption, requiring a Greenhouse-Geisser correction. The simple effect of gender ratio was significant for male observers, *F*_(1.18, 42.59)_ = 17.51, *p* < 0.001, η^2^_*p*_ = 0.33, but not for female observers, *p* > 0.05. Simple contrasts demonstrated that men looked at individual female pictures longer when the female-to-male gender ratio was 3:1 vs. 4:0, 1:3 vs. 4:0, or 1:3 vs. 3:1. In addition, simple effects examining gender differences in gaze duration within each four-picture trial demonstrated a significant effect of gender when women were the minority 1:3, *t*_(60.04)_ = 2.84, *p* = 0.006, such that male observers looked at female pictures more than female observers. Male gazers looked at individual female pictures for longer when fewer female pictures were in the trial.

A similar pattern was seen when comparing time female pictures were viewed during six-picture trials. A 2 (observer gender) × 5 (gender ratio) mixed-design ANOVA demonstrated significant main effects of observer gender, *F*_(1, 74)_ = 11.97, *p* < 0.001, η^2^_*p*_ = 0.14, and gender ratio *F*_(4, 296)_ = 17.52, *p* < 0.001, η^2^_*p*_ = 0.19, and an interaction between observer gender and gender ratio, *F*_(4, 296)_ = 7.09, *p* < 0.001, η^2^_*p*_ = 0.09. Two repeated-measures ANOVAs analyzed the simple effects of trial among either female or male gazers. Based on gender ratio, there were no significant differences in how long female gazers observed individual female pictures (*p* > 0.05). For the within-subjects ANOVA examining the effect of gender ratio on male gaze toward female pictures the sphericity assumption was violated necessitating a Greenhouse-Geisser correction. That analysis revealed a significant simple effect of gender ratio for male gazers, *F*_(2.03, 73.13)_ = 15.28, *p* < 0.001, η^2^_*p*_ = 0.30. Simple contrasts demonstrated that men viewed individual female pictures longer when fewer female pictures were displayed across all six-picture trial types, *p* < 0.05, except trials with female-to-male ratios of 5:1 vs. 4:2, *p* > 0.05. Independent *t*-tests evaluated the simple effects of observer gender within each six-picture condition. There were significant differences in gaze duration based on observer gender within all six-picture trials, *p* < 0.05, except within the baseline trial containing only female pictures. The fewer number of female pictures in a six-picture condition, the longer a given female picture was viewed, particularly by male gazers.

#### Male gender ratio

The opposite effect was found for male pictures, such that male pictures were viewed less when fewer pictures of men were in the group. A 2 (observer gender) × 2 (gender ratio) mixed-design ANOVA analyzed the average time female and male gazers attended to male pictures in two-picture trials with different gender ratios. In two-picture trials with either one male and one female picture or two male pictures, there were significant main effects of observer gender, *F*_(1, 74)_ = 5.14, *p* = 0.03, η^2^_*p*_ = 0.07, and gender ratio, *F*_(1, 74)_ = 11.01, *p* = 0.001, η^2^_*p*_ = 0.13, and an interaction between the two, *F*_(1, 74)_ = 6.33, *p* = 0.01, η^2^_*p*_ = 0.08. Female observers viewed male pictures longer than men overall, though in general male pictures were looked at less with only one male picture on the screen.

A 2 (observer gender) × 3 (gender ratio) ANOVA examining gaze duration toward men in four-picture trials with varying gender ratios demonstrated a significant main effect of observer gender, *F*_(1, 74)_ = 11.38, *p* = 0.001, η^2^_*p*_ = 0.13) and gender ratio, *F*_(2, 148)_ = 28.76, *p* < 0.001, η^2^_*p*_ = 0.28. The interaction between observer gender and gender ratio was non-significant, *p* > 0.05. Female gazers viewed male pictures for longer periods of time than male gazers, and male pictures were looked at less when fewer male pictures were shown. This pattern was seen across six-picture trials as well. A 2 (observer gender) × 5 (gender ratio) ANOVA using a Greenhouse-Geisser correction for violation of the sphericity assumption revealed significant main effects of observer gender, *F*_(1, 74)_ = 6.36, *p* = 0.01, η^2^_*p*_ = 0.08, and gender ratio, *F*_(2.14, 158.64)_ = 7.26, *p* < 0.001, η^2^_*p*_ = 0.09, and a non-significant interaction, *p* > 0.05. Overall, women looked at male pictures longer than men. *Post-hoc* tests demonstrated significant differences in gaze duration between trials with male-to-female ratios of 6:0 vs. 5:1, 6:0 vs. 2:4, 6:0 vs. 1:5, 5:1 vs. 2:4, and 4:2 a vs. 2:4, *p* < 0.05. Overall, male pictures were looked at for shorter amounts of time when fewer male pictures were in the trial.

### Gaze frequency

The number of times women's and men's pictures were viewed in each trial was averaged for individual observers in order to examine frequency of gaze directed toward women or men. It was hypothesized that observers would view pictures of women more frequently, as compared to pictures of men. A 2 (observer gender) × 2 (target gender) mixed-design ANOVA assessed differences in gaze frequency toward female and male pictures across trials, based on observer gender. There was a main effect of picture gender, *F*_(1, 74)_ = 58.17, *p* < 0.001, η^2^_*p*_ = 0.44, though observer gender and the interaction term were non-significant, *p* > 0.05. Female pictures (*M* = 3.45, *SD* = 0.89) were looked at significantly more times per trial than male pictures (*M* = 3.12, *SD* = 0.84).

A series of planned comparisons further analyzed differences in the frequency of looks by women and men observers toward pictures of women and men in trials with similar gender ratios (e.g., 1:5 female-to-male and 1:5 male-to-female). The majority of these comparisons were non-significant, *p* > 0.05, with the exception of two trial types: groups with a 2:4 minority and 1:5 minority. An ANOVA comparing the frequency of gaze toward female and male pictures in a 2:4 minority revealed a significant main effect of condition, *F*_(1, 74)_ = 39.80, *p* < 0.001, η^2^_*p*_ = 0.35. Female and male gazers looked at female pictures more frequently than male pictures when there were two vs. four such pictures in the trial (*M* = 2.92, *SD* = 1.05; *M* = 2.14, *SD* = 0.59). Examining this effect in a 1:5 minority also demonstrated a significant main effect of gender ratio, *F*_(1, 74)_ = 15.10, *p* < 0.001, η^2^_*p*_ = 0.17, as well as an interaction between observer gender and gender ratio, *F*_(1, 74)_ = 6.02, *p* = 0.02, η^2^_*p*_ = 0.08. The main effect of observer gender was non-significant (*p* > 0.05). Men viewed individual female pictures more times (*M* = 3.17, *SD* = 0.80) and male pictures fewer times (*M* = 2.36, *SD* = 0.93) than women observers (*M* = 2.63, *SD* = 1.09; *M* = 2.44, *SD* = 0.86).

### Gaze sequence

It was predicted that pictures of women would be more likely to be viewed first and last across trials, demonstrating observers' immediate and ongoing focus on female targets over male targets. A 2 (observer gender) × 2 (target gender) mixed-design ANOVA was conducted to determine whether female or male pictures were more likely to be looked at during the first five gaze samplings. There was a significant effect of target gender, *F*_(1, 74)_ = 25.38, *p* < 0.001, η^2^_*p*_ = 0.26, though observer gender and the interaction between observer gender and target gender were non-significant, *p* > 0.05. Female pictures were more likely to be viewed during the first five gaze samplings, as compared to male pictures (*M* = 94.04, *SD* = 7.26; *M* = 89.08, *SD* = 7.33).

A 2 × 2 ANOVA analyzing the last five gaze sequences across trials demonstrated a non-significant effect of observer gender, *p* > 0.05, a significant effect of target gender, *F*_(1, 74)_ = 8.62, *p* = 0.004, η^2^_*p*_ = 0.10, and a significant interaction between the two, *F*_(1, 74)_ = 4.25, *p* = 0.04, η^2^_*p*_ = 0.05. Women and men looked at pictures of women more than pictures of men in the last five sequence points. In addition, women looked at male pictures (*M* = 111.10, *SD* = 6.90) more than men did (*M* = 107.59, *SD* = 7.55), and men looked at female pictures (*M* = 114.19, *SD* = 6.87) more than women observers did (*M* = 112.26, *SD* = 6.95).

### Gaze avoidance

A series of mixed-design ANOVAs compared women and men's gaze avoidance for each trial type with the same number of pictures. It was hypothesized that gazers would demonstrate less gaze avoidance during trials with more pictures of women. This hypothesis was not supported. Female and male observers looked at pictures and blank space for approximately the same duration regardless of the gender composition. This was true across trials with one, two, and four pictures, *p* > 0.05. Among six-picture trials, a 2 (observer gender) × 6 (gender ratio) ANOVA demonstrated non-significant main effects of observer gender and gender ratio, *p* > 0.05, but a significant interaction between gender and picture composition, *F*_(5, 370)_ = 2.40, *p* = 0.04, η^2^_*p*_ = 0.03. Simple-effects analysis revealed no significant comparisons within gender or trials, *p* > 0.05. Overall, women had somewhat higher levels of gaze avoidance when viewing groups with a female majority (*M* = 0.17, *SD* = 0.13) than a male majority (*M* = 0.16, *SD* = 0.13), and men displayed more gaze avoidance when viewing majority male groups (*M* = 0.17, *SD* = 0.13) than female majority groups (*M* = 0.15, *SD* = 0.12).

An additional 2 (observer gender) × 4 (picture number) ANOVA compared women's and men's average gaze avoidance across trials with either one, two, four, or six pictures. The main effects of observer gender and picture number, and the interaction term, were all non-significant, *p* > 0.05. Regardless of how many pictures were on the screen, gazers looked at blank space vs. pictures for approximately equal amounts of time. Lastly, a *t*-test comparing women's and men's average gaze avoidance across all trials revealed that average gaze avoidance was not significantly different based on gender, *p* > 0.05. Observers viewed pictures vs. blank screen space for approximately equal amounts of time regardless of their gender, the gender ratio of pictures, or the number of the pictures presented to them.

### Beliefs about social gaze

Survey questions were used to investigate the relation between actual social gaze behavior and self-reported gaze behavior, with the hypothesis that male observers would be more accurate than female observers in predicting their own gendered gaze behavior. A mixed-design 2 (observer gender) × 2 (target gender) ANOVA compared women's and men's self-reported beliefs about their own gazing behavior toward people of the same- and opposite-sex. There was a significant main effect of target gender, *F*_(1, 74)_ = 12.84, *p* = 0.001, η^2^_*p*_ = 0.15, but not of observer gender, *p* > 0.05. There was also an interaction between observer gender and target gender, *F*_(1, 74)_ = 186.52, *p* < 0.001, η^2^_*p*_ = 0.72. Female gazers reported looking at men more than women (*M* = 5.00, *SD* = 1.56; *M* = 2.82, *SD* = 1.65), and male gazers reported looking at women more than men (*M* = 5.46, *SD* = 0.93; *M* = 1.73, *SD* = 1.26).

A 2 × 2 ANOVA evaluated observers' beliefs about how much they are looked at by other men and women. There were significant main effects of target gender, *F*_(1, 74)_ = 5.59, *p* = 0.02, η^2^_*p*_ = 0.07, and observer gender, *F*_(1, 74)_ = 4.71, *p* = 0.03, η^2^_*p*_ = 0.06, as well as a significant interaction between target gender and observer gender, *F*_(1, 74)_ = 98.05, *p* < 0.001, η^2^_*p*_ = 0.57. Women reported that they are looked at more by men than women (*M* = 4.62, *SD* = 1.35; *M* = 3.23, *SD* = 1.40), while men reported that they are looked at more by women than men (*M* = 4.43, *SD* = 1.02; *M* = 2.27, *SD* = 1.43).

Observers were also asked to what extent they pay attention to attractive and unattractive people. A 2 (observer gender) × 2 (target attractiveness) mixed-design ANOVA evaluated gender differences in self-reported gazing behavior toward “attractive” and “unattractive” people. There was a significant main effect of attractiveness of target, *F*_(1, 74)_ = 163.59, *p* < 0.001, η^2^_*p*_ = 0.69, and a significant interaction between attractiveness of target and gender of gazer, *F*_(1, 74)_ = 5.67, *p* = 0.02, η^2^_*p*_ = 0.07. The main effect of observer gender was not significant, *F*_(1, 74)_ = 1.79, *p* = 0.19, η^2^_*p*_ = 0.02. Women reported looking at unattractive people more than men (*M* = 3.46, *SD* = 1.30; *M* = 3.24, *SD* = 1.50), and men reported looking at attractive people more than women (*M* = 6.27, *SD* = 0.77; *M* = 5.54, *SD* = 1.14). On average, women and men reported looking at attractive people more than unattractive people.

## Discussion

Objectification theory suggests the evaluation of women, particularly by men, is ubiquitous and that a stranger's gaze can trigger women to become vigilant to their own appearance and self-objectify (Fredrickson and Roberts, 1997). The present study is the first to test the hypothesis that women are looked at more than men by examining visual attention in groups of varying gender composition. This study is also among the first to examine objectifying gaze and visual attention in social groups using eye tracker technology.

In line with objectification theory, gender played a significant role in determining attention to different social targets. In mixed-gender trials men and women observers visually evaluated women more than male targets. Women were viewed more frequently and for longer periods of time than men in mixed-gender groups, and they were likely to be looked at first and last by female and male observers. Men, in turn, were viewed for shorter periods of time in mixed-gender groups and less frequently overall than their female counterparts. Men's preference for looking at women was magnified when women were the minority. Men spent more time attending to women when fewer were in the group. The opposite was true when it came to viewing men, such that when fewer men were shown observers directed less attention toward them. In addition, female observers spent more time looking at men as compared to male observers and male observers spent more time looking at women than female observers, though overall female and male observers looked at women more than men. Women's appearance garners more attention and interest in mixed-gender social groups.

In contrast, comparing baseline trials with either all female or all male pictures, pictures of women and men were equally likely to be observed. This is accounted for by the finding that, regardless of the number of pictures presented or the picture composition, observers spent relatively equal lengths of time attending to pictures of people vs. blank screen space. Men and women had a similar baseline interest in viewing others, contradicting previous findings that women display more eye contact during social interactions, including gaze (Hall, 1984).

### Implications

Social gaze may act as a nonverbal signal of sexualization, dominance, social comparison, or desire for closeness, among other things (Hall, 1963; Exline et al., 1975; Fredrickson and Roberts, 1997; Gervais et al., 2013). For heterosexual men, gaze toward women has been discussed in terms of sexual interest and dominance. It is possible that men may have a stronger preference for viewing the opposite-sex because appearance is a stronger determinant of sexual interest for men. Theories of human sexuality suggest that men often select mates based on physical signs of fertility. Women rely on physical traits to some extent when choosing a partner, but place more weight on qualities such as status and intelligence (e.g., Buss and Schmitt, 1993; Gangestad and Simpson, 2000). Male observers, particularly young adults like those in the current sample, may scan social groups to identify attractive partners.

In scanning for attractive others, attention does not necessarily lead to approach behavior (e.g., Gruenfeld et al., 2008). Social psychological theory suggests that “girl watching,” even in the absence of sexual intent, is strongly associated with masculinity and is a means of denying women's other identities and maintaining asymmetrical power relationships. Visually monitoring women may be a means of patrolling traditional gender boundaries (Rosenberg, 1988; Fredrickson and Roberts, 1997; Quinn, 2002). Considering that “sexual harassment” is defined by “unwanted gender-related attention” (Kelly et al., 2005, p. 689; Fitzgerald et al., 2002) and “sexism” by the “inhibition of women through a vast network of everyday practices” (Young, 1992, p. 180; Lott, 1995), the present study suggests that small acts of bias in the form of gendered gaze are commonplace.

Men and women were similarly more interested in viewing women than men, corroborating previous work that shows both men and women objectify women through gaze (Heflick et al., 2011; Vaes et al., 2011; Bernard et al., 2012; Gervais et al., 2013). Women's interest in viewing other women also has implications for social comparison theory. This theory states that, in the absence of objective evaluative criteria, people evaluate themselves by making comparisons with others, especially those who share relevant characteristics (Festinger, 1954; Gervais et al., 2013). Female gazers may have been more focused on evaluating themselves against other women than viewing pictures of men. This preference may reflect the process of self-objectification whereby women are vigilant to how they measure up to others in terms of physical appearance.

Visual attention toward women may indicate a desire for interpersonal closeness rather than a process of women's objectification. Along these lines, women tend to exhibit a stronger automatic in-group orientation than men. Favoritism toward women is associated with stereotypes that deem women as warmer, but weaker and less intimidating, than men (Rudman and Goodwin, 2004). People may be more likely to approach women because they are seen as relationship-oriented and non-threatening. However, the present study only provided observers with images of women and men, and a focus on women's outward appearance tends to increase objectification and decrease perceptions of women's competence, warmth, and morality (Heflick et al., 2011). Therefore, the findings are consistent with the hypothesis that women are attractive targets of gaze because of the greater emphasis placed on women's appearance, not solely due to stereotypes of women as relationship-oriented.

Findings that women's salience in social groups increases when they are the minority has implications for women in traditionally male-dominated domains such as leadership and Science, Technology, Engineering, Mathematics, and Medicine (STEMM). In professional settings, minority status can increase STEMM women's physiological arousal and recall of the environment, and reduce comfort when attending professional events (e.g., Murphy et al., 2007). Potential for visual evaluation in these environments may increase discomfort, as objectifying gaze impacts women's performance in the STEMM disciplines (Gervais et al., 2011). Rather than being overlooked in male-dominated domains, women may be monitored more closely. This is consistent with previous findings that solo status increases gender salience (Taylor et al., 1978).

Male participants were more accurate than female participants in reporting their gaze behavior. Men accurately reported that they looked at women more than men, and men reported that their visual attention was often driven by target attractiveness. Women inaccurately reported that they looked at men more than they looked at women. Men's readiness to share that they gaze at women more than men reflects that male gaze is accepted as a heterosexist norm, and it is assumed that heterosexual men will “check out” the women around them. Women, on the other hand, may not realize that they may similarly objectify women through physical evaluation.

### Limitations

The present study is limited in removing common contextual variables that determine gaze behavior, for example, clothing style, social setting, interpersonal relationships, and potential for mutual gaze. While results cannot be generalized across all social contexts, they are useful for understanding baseline preferences for viewing men and women. In addition, trials exposed people to unfamiliar faces for brief amounts of time. Thin slices of time are sufficient for gathering information about social targets, including face gender recognition and more specific inferences about personality traits (e.g., Ito and Urland, 2003; Carney et al., 2007). Baltazar et al. (2014) have also established that eye contact during brief exposure to faces is enough to trigger bodily self-awareness, similar to that seen in self-objectification. However, more research is needed to determine how gaze behavior evolves over time and in different contexts. Another limitation is the examination of gaze behavior between primarily White, young adults enrolled in college. Certainly, viewing preferences may differ between people of different social backgrounds or change over the lifespan. Additional research must investigate this possibility.

Lastly, it is unclear what factors contribute to the negative and positive effects of gaze for targets. According to objectification theory, gender biases in gaze behavior may disproportionately and negatively affect women. On the other hand, in some circumstances the absence of visual attention may be an indicator of disinterest or even ostracism, which comes with its own negative effects (Williams, 2007). Research in this area may offer insight on how to reduce the consequences of gendered gaze, which the present study has shown to occur with relative frequency.

## Concluding remarks

Women are looked at for longer periods of time in mixed-gender groups, particularly when they are the minority, and they are likely to be looked at first, last, and more frequently by men and women gazers. In the absence of information to the contrary, social gaze demonstrates visual inspection and the potential for objectification (Kaschak, 1992; Fredrickson and Roberts, 1997), a process linked with negative physical, psychological, and social outcomes (see Calogero et al., 2011, for a review). The present study helps demonstrate the normative experience of women, and calls for more interventions to ameliorate the influence of these many small acts of gender bias.

### Conflict of interest statement

The author declares that the research was conducted in the absence of any commercial or financial relationships that could be construed as a potential conflict of interest.
